# Lysine demethylase LSD1 is associated with stemness in EBV-positive B cell lymphoma

**DOI:** 10.1038/s41598-024-55113-6

**Published:** 2024-03-21

**Authors:** Joo Hyun Kim, Chaehwa Park, Won Seog Kim

**Affiliations:** 1 Department of Health Sciences and Technology, Samsung Advanced Institute for Health Sciences and Technology, Sungkyunkwan University, Seoul, 06351 Korea; 2 Research Institute for Future Medicine, Samsung Medical Center, Sungkyunkwan University School of Medicine, Seoul, 06351 Korea; 3 Division of Hematology and Oncology, Department of Medicine, Samsung Medical Center, Sungkyunkwan University School of Medicine, 50 Irwon-dong, Seoul, 06351 Korea

**Keywords:** Cancer, Drug discovery, Molecular medicine

## Abstract

EBV-infected lymphoma has a poor prognosis and various treatment strategies are being explored. Reports suggesting that B cell lymphoma can be induced by epigenetic regulation have piqued interest in studying mechanisms targeting epigenetic regulation. Here, we set out to identify an epigenetic regulator drug that acts synergistically with doxorubicin in EBV-positive lymphoma. We expressed the major EBV protein, LMP1, in B-cell lymphoma cell lines and used them to screen 100 epigenetic modifiers in combination with doxorubicin. The screening results identified TCP, which is an inhibitor of LSD1. Further analyses revealed that LMP1 increased the activity of LSD1 to enhance stemness ability under doxorubicin treatment, as evidenced by colony-forming and ALDEFLUOR activity assays. Quantseq 3′ mRNA sequencing analysis of potential targets regulated by LSD1 in modulating stemness revealed that the LMP1-induced upregulation of CHAC2 was decreased when LSD1 was inhibited by TCP or downregulated by siRNA. We further observed that SOX2 expression was altered in response to CHAC2 expression, suggesting that stemness is regulated. Collectively, these findings suggest that LSD1 inhibitors could serve as promising therapeutic candidates for EBV-positive lymphoma, potentially reducing stemness activity when combined with conventional drugs to offer an effective treatment approach.

## Introduction

Epstein–Barr Virus (EBV) infects B cells and regulates their growth, survival, and differentiation; this can occur through epigenetic regulation, which results in B cell transformation and lymphomagenesis^1–4^. EBV-positive B cell lymphomas are heterogeneous and have a poor prognosis; thus, there is urgent need to identify druggable targets and thereby improve the therapeutic efficiency and patient survival^1,4^. Recent reports indicating that various types of epigenetic regulation can induce and promote B lymphoma have sparked interest in the mechanism(s) underlying the ability of EBV infection to modulate epigenetic regulation^2^. Aberrant epigenetic regulations, including dysregulation of DNA methylation and histone modification, have been established as cancer hallmarks and valuable therapeutic targets. Epigenetic regulators have been identified as oncogenes or tumor suppressor genes involved in cancer initiation, progression, and therapeutic failure^5^. Given the reversible nature of epigenetic alterations, there is significant interest in targeting epigenetic modulators to treat cancer^6^. The epigenetic modifier, lysine-specific demethylase 1 (LSD1/KDM1A), is a flavin adenine dinucleotide (FAD)-dependent amine oxidase family member that is responsible for demethylating monomethyl or dimethyl lysine 4 (K4) on histone H3. LSD1 has garnered significant attention for its role in regulating embryonic stem cells (ESCs) and its relevance in cancer research. It is upregulated in numerous malignancies, particularly in aggressive and poorly differentiated tumors^7–10^. Considering the pivotal role of LSD1 in stem cell properties, its dysregulation in cancer could potentially impact pathways associated with a stem cell-like phenotype. Certainly, recent studies across diverse cancer types have supported the idea that LSD1 has a fundamental regulatory function within cancer stem cells (CSCs), highly aggressive subgroup of tumors characterized by their unique properties. LSD1 can also regulate transcription, either activating or repressing genes depending on the context and interactions with specific partners or multiprotein complexes^11^. LSD1 has been further implicated in various biological processes, including the DNA damage response, apoptosis, DNA methylation, and angiogenesis, and been shown to act on non-histone substrates, such as E2F-1, DNMT1, MYPT1, HIF-1α, and STAT3^12–16^. Tranylcypromine (TCP), which is a monoamine oxidase (MAO) inhibitor employed in clinical practice to treat depression^17^, was identified as a reversible and relatively mild inhibitor of LSD1^18,19^. Researchers are currently assessing the therapeutic efficacy of TCP against AML and MDS, alone or in combination with ATRA. The blockade of LSD1 with iadademstat has also emerged as a promising strategy for addressing certain solid tumors, such as small-cell lung cancer (SCLC) and melanoma^20–22^. Recent studies have suggested that the iadademstat-mediated inhibition of LSD1 activity may stimulate immune responses, potentially offering a novel approach for overcoming resistance to immune checkpoint inhibitors in melanoma^22^. CHAC2, which belongs to the cation transport regulator-like protein (CHAC) family, contributes to glutathione degradation and thereby impacts the cellular redox potential^23^. CHAC2 is crucial for the ability of human embryonic stem cells (hESCs) to maintain pluripotency and is prominently expressed in undifferentiated hESCs and induced pluripotent stem cells (iPSCs)^24^. Overexpression of wild-type CHAC2, but not mutant CHAC2, significantly enhances the proliferative capacity of breast cancer cells^25^. Additionally, CHAC2 activates the MAPK signaling pathway and promotes the development of lung adenocarcinoma^26^. These previous findings collectively indicate that CHAC2 plays a complex role in GSH metabolism and, through its enzymatic activity, significantly impacts the progression of tumors. Here, we aimed to select epigenetic regulators that showed synergistic growth inhibition effects with doxorubicin in EBV-positive lymphoma cells, and to observe the function of these modifiers in the presence of LMP1. From the obtained results, we sought to identify a target gene that could be developed into an effective treatment method for EBV-positive lymphoma.

## Results

### Epigenetic modifier screen identifies TCP as acting synergistically with doxorubicin in LMP1-expressing B cell lymphoma cells

**Figure 1:**
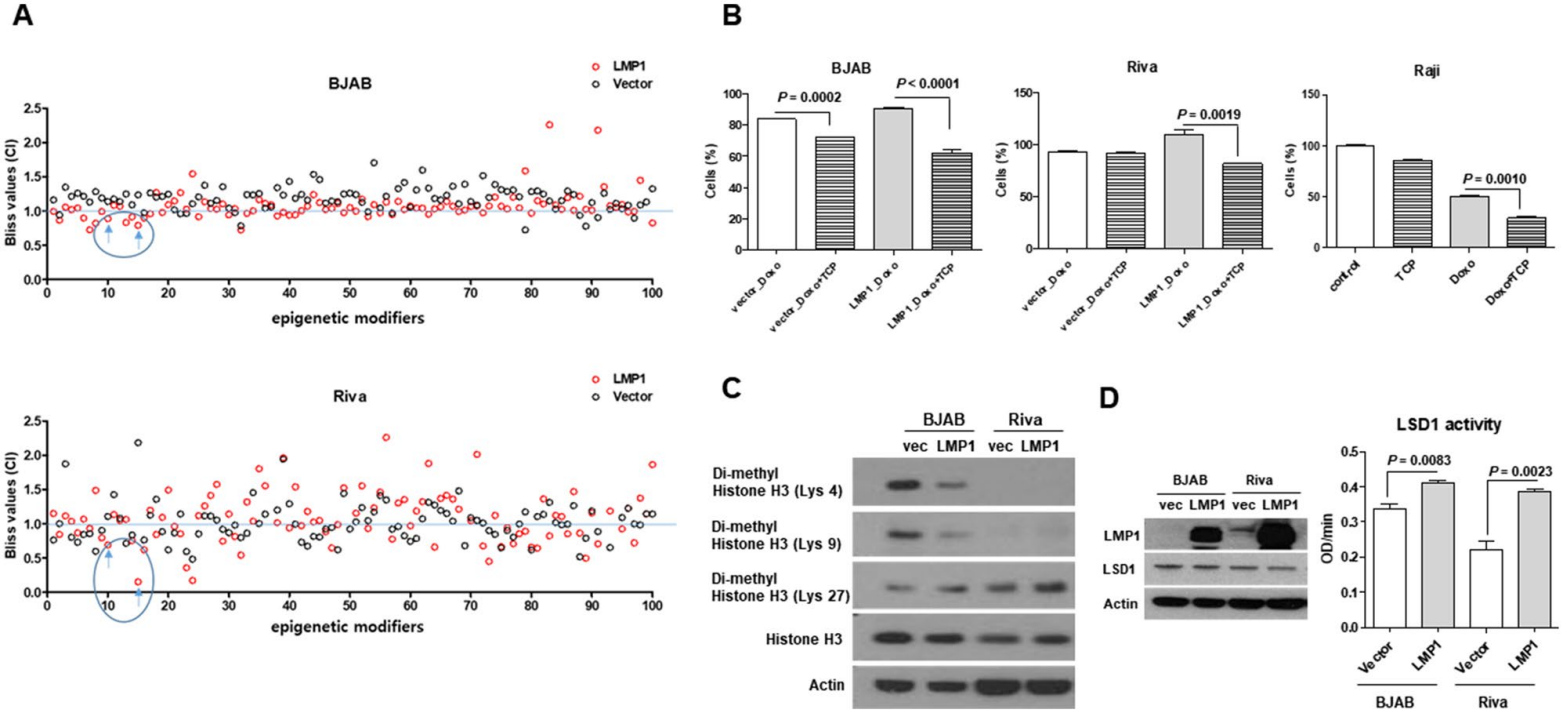
LSD1 inhibition increases the ability of doxorubicin to inhibit cell viability in LMP1-expressing B cell lymphoma cells. (**A**) Screening of 100 epigenetic modifiers plus doxorubicin against B cell lymphoma cell lines with and without expression of LMP1. Cells were treated with each epigenetic modifier (300 nM) plus doxorubicin (BJAB_30nM, Riva_100nM) for 48 h and cell viability was measured using the CCK-8 assay. Epigenetic modifiers that showed synergistic effects in combination with doxorubicin are shown with their Combination Indexes (Bliss values). Blue arrows indicate epigenetic modifiers that showed a common synergistic effect in BJAB and Riva cells by calculating CI when LMP1 is expressed compared to vector. (**B**) BJAB and Riva cell viability was measured by trypan blue staining. Cells were harvested after 48 h of treatment with the indicated drugs. P-values were determined by the Student’s *t*-test. Cells (%) was calculated relative to control and LMP1-expressing cells that were not treated with doxorubicin or TCP. (**C**) Western blot analysis of histone H3 modification levels in LMP1-expressing BJAB and Riva cells compared to control cells. Blots cropped from different gels were grouped together because the protein size was the same. (**D**) LSD1 expression and activity in LMP1-expressing BJAB and Riva cells compared to control cells.

Given the inherently reversible nature of epigenetic alterations, epigenetic modulators are considered to be promising therapeutic targets against cancer^27^. Doxorubicin, a widely used first-line chemotherapeutic agent for B cell lymphoma, served as the standard drug. In our efforts to identify epigenetic modifiers that could enhance the inhibition of cell proliferation in EBV-positive B cell lymphoma when combined with doxorubicin, we initially established EBV-positive B cell lymphoma cell lines using two B-cell lymphoma cell lines: BJAB cells and Riva cells. These cell lines were transfected with pEGFP-N1-LMP1, which is the major signal transduction protein of EBV. We confirmed by IF staining that LMP1-expressing cell lines were stably established, with LMP1 expressed in more than 80–90% of the cells (Supple Fig. 1). Next, we sought to identify epigenetic modifiers that could work in synergy with doxorubicin to decrease the proliferation of the LMP1-expressing B cell lymphoma cells. In cell lines expressing vector and LMP1, the combination index (CI) value was calculated and graphed; it was based on the inhibition values obtained from cells treated with doxorubicin plus epigenetic modifiers versus those treated with doxorubicin alone. As shown in Fig. 1A, calculation of the combination index (CI) values for doxorubicin in combination with each of 100 epigenetic modifiers revealed that TCP (second blue arrow) had synergistic effects with doxorubicin in BJAB and Riva cells. The results of our trypan blue exclusion assay confirmed the synergistic effects of TCP and doxorubicin in LMP1-expressing B cell lymphoma cell lines (Fig. 1B). Interestingly, synergistic effects of doxorubicin with TCP were mainly observed in LMP1-expressing cells. These results were also confirmed in Raji cells, which represent an EBV-positive B cell lymphoma cell line (Fig. 1B). Next, we assessed whether LMP1 regulates the expression and/or activity of LSD1. As shown in Fig. 1C, measurement of histone methylation levels showed that LMP1 decreased the levels of H3K4me2 and H3K9me2, while that of H3K27me2 was increased. We also found that the activity of LSD1 was increased in LMP1-expressing cell lines, whereas its expression was unchanged (Fig. 1D). These data suggest that LMP1 may modulate the sensitivity of EBV-positive B cell lymphoma cells to epigenetic modifiers by regulating the activity of LSD1.

### LSD1 induces stemness and regulates apoptosis

**Figure 2:**
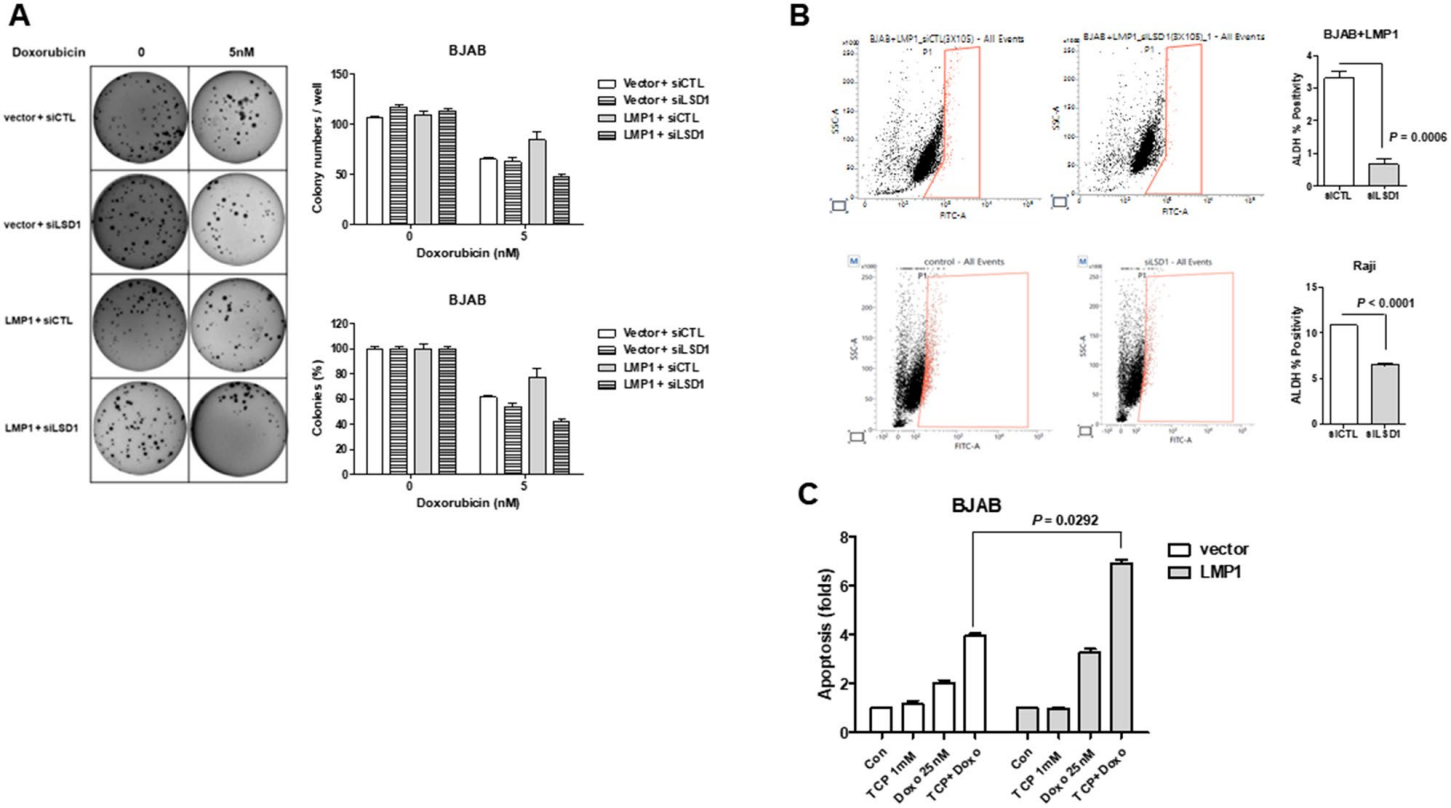
LSD1 regulates the stemness-associated activity of LMP1-expressing BJAB cells. (**A**) Colony-formation assays were performed using control or LMP1-expressing BJAB cells transfected with siCTL or siLSD1 and treated with 5 nM doxorubicin. The data are presented as the mean ± standard deviation; n = 3. Bar graph presents colony numbers/well or colony (%) compared to untreated cells. P-values were determined by the Student’s *t*-test. (**B**) FACS analysis of ALDH-positive cells in control and LMP1-expressing BJAB cells and EBV-positive Raji cells. Representative plots and summary data showing the percentage of ALDH1-positive control cells, LMP1-expressing BJAB cells, and EBV-positive Raji cells. P-values were determined by the Student’s *t*-test. (**C**) Percentages of apoptotic cells in control and LMP1-expressing cell cultures treated for 72 h with TCP (1 mM) and doxorubicin (25 nM) alone or in combination, as measured by Annexin V/PI assay. The data are presented as the mean ± standard deviation; n = 3. P-values were determined by the Student’s *t*-test.

**Figure 3:**
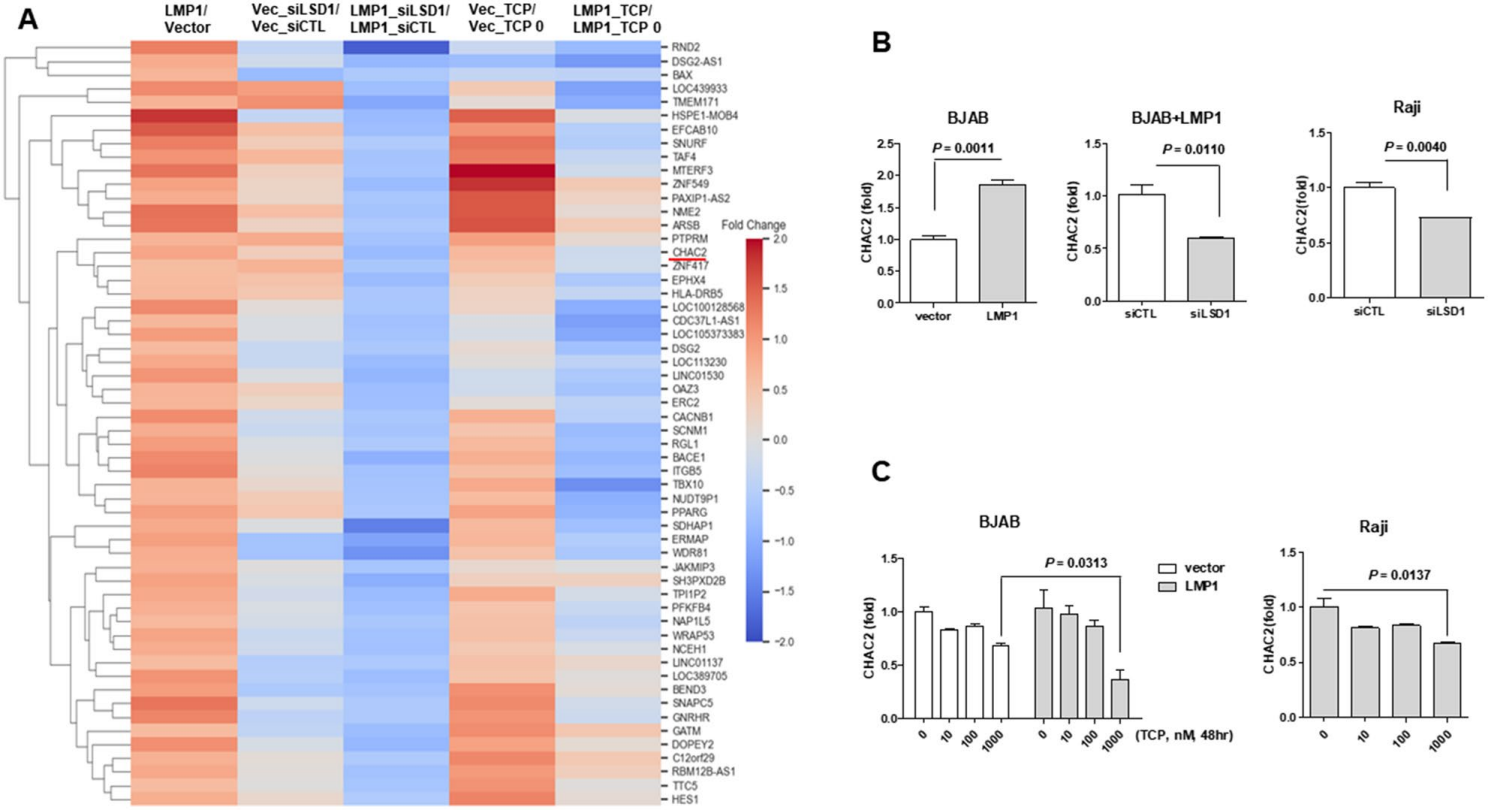
Genes showing differential expression following LSD1 inhibition in control versus LMP1-expressing BJAB cells. (**A**) Cells were transfected with siCTL or siLSD1 and treated with TCP (1 mM) for 48 h. QuantSeq 3′ mRNA-sequencing analysis was performed to identify mRNAs that were downregulated by both TCP treatment and siLSD1 transfection in LMP1-expressing cells compared to control cells. (**B**) LMP1-expressing BJAB cells and EBV-positive Raji cells were transfected with siRNA control or siLSD1 for 48 h and the mRNA expression levels of CHAC2 were detected by qRT-PCR. (**C**) Control or LMP1-expressing BJAB cells and EBV-positive Raji cells were treated with various concentrations of TCP for 48 h, and the mRNA expression levels of CHAC2 were detected by qRT-PCR. P-values were determined by the Student’s *t*-test.

**Figure 4:**
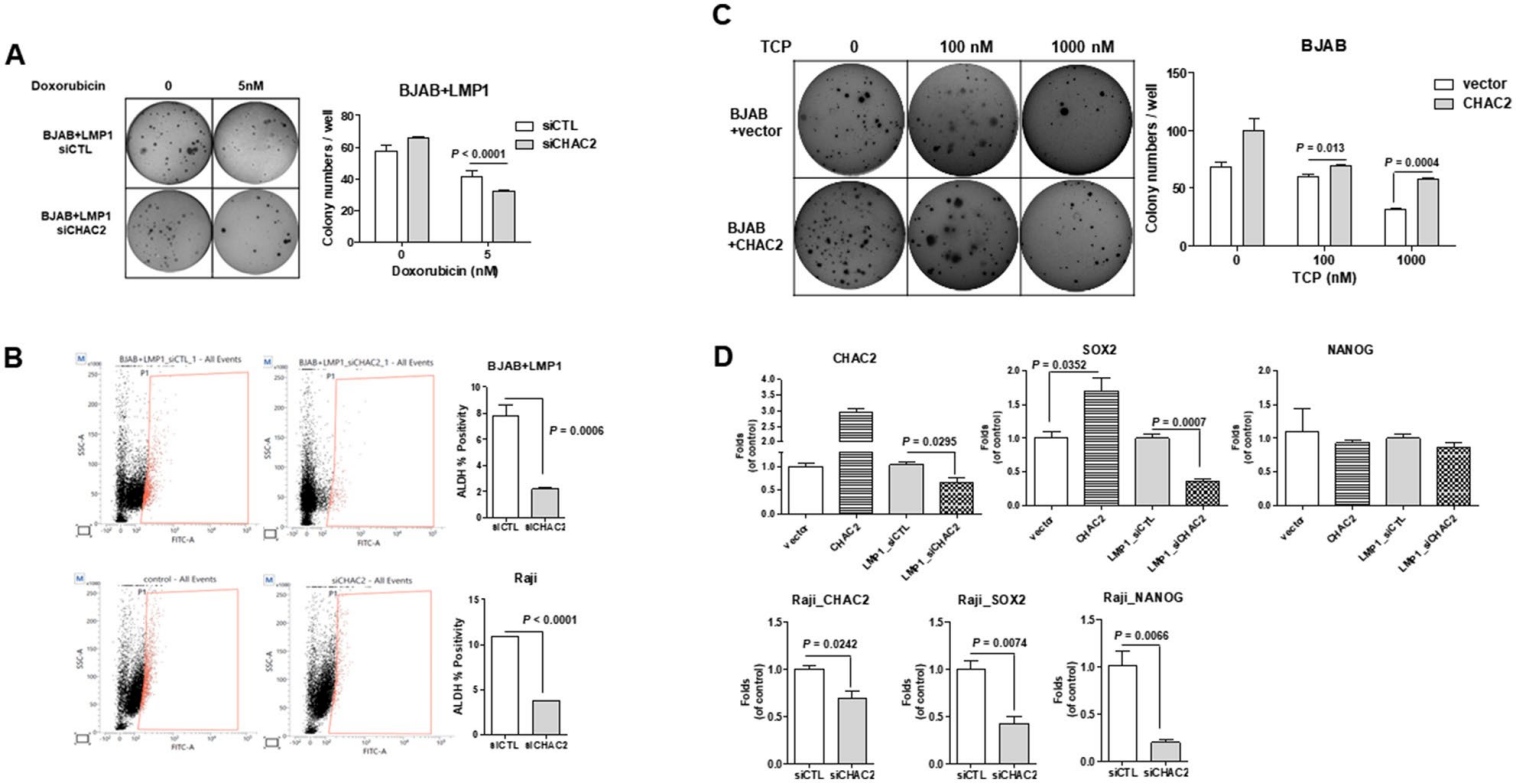
LSD1-regulated CHAC2 mediates stemness in LMP1-expressing BJAB cells. (**A**) Colony-formation assays were performed using LMP1-expressing BJAB cells transfected with siCTL or siCHAC2 and treated with 5 nM doxorubicin. The data are presented as the mean ± standard deviation; n = 3. Bar graphs present colony numbers/well or colonies (%) compared to untreated cells. P-values were determined by the Student’s *t*-test. (**B**) FACS was used to analyze ALDH-positive cells among LMP1-expressing BJAB cells and EBV-positive Raji cells transfected with siCTL or siCHAC2. Representative plots and summary data showing the expression of ALDH1 according to CHAC2 knockdown in control and LMP1-expressing BJAB cells. P-values were determined by the Student’s *t*-test. (**C**) Colony-formation assays were performed using BJAB cells transfected with pCMV6-entry control vector or CHAC2 and treated with 100 nM or 1000 nM TCP. Bar graphs present colony numbers/well or colony (%) compared to untreated cells. (**D**) BJAB cells were transfected with pCMV6-entry control vector or CHAC2 and LMP1-expressing BJAB cells were transfected with siCTL or siCHAC2. EBV-positive Raji cells transfected with siCTL or siCHAC2. The mRNA expression levels of CHAC2, SOX2, and NANOG were detected by qRT-PCR. P-values were determined by the Student’s *t*-test.

LSD1 is known to stimulate cell proliferation, promote stemness in various cancer^28^. To further examine the effect of LSD1 expression on stemness in our system, we used siRNA to suppress LSD1 expression in LMP1-expressing cells. As demonstrated in Fig. 2A, LSD1 knockdown reduced the colony-forming ability of doxorubicin-treated LMP1-expressing BJAB cells, although no difference was observed in the absence of doxorubicin. Moreover, the spheroid forming rate was low in LSD1-inhibited LMP1-expressing cells. However, the spheroid diameter was around 100 μm in both cell lines (Supple Fig. 2A). To further monitor the presence of CSCs, we used the ALDEFLUOR assay to detect the activity of ALDH1. Indeed, the ALDEFLUOR-positive population was upregulated by LSD1 in LMP1-expressing BJAB cells and EBV-positive Raji cells (Fig. 2B). Since the proportion of CSCs is a principal determinant of the response to chemotherapy, we examined whether LSD1 could affect the drug sensitivity of lymphoma cells. Our results showed that treatment with TCP plus doxorubicin was more effective than doxorubicin alone (Supple Fig. 3A) and induced more apoptosis in LMP1-expressing cells than control cells (Fig. 2C, Supple Fig. 3B). Decreased expression of anti-apoptotic MCL-1 was observed in Western blot analysis (Supple Fig. 3C). To explore the mechanism underlying the synergistic activity of TCP plus doxorubicin in LMP1-expressing cells, we performed cDNA microarray analysis (Supple Fig. 4A). As shown in Supplementary Fig. 4B, the expression levels of MMP9 and ROR2 were increased in LMP1-expressing cells under combined treatment with TCP and doxorubicin. Conversely, when MMP9 and ROR2 expression was inhibited using siRNA, colony formation was increased (Supple Fig. 4C). Therefore, increased expression of MMP9 and ROR2 may play important roles in the survival of lymphoma cells following treatment with TCP plus doxorubicin.

### LSD inhibition regulates CHAC2-mediated stemness

LSD1 is involved in multiple biological aspects of cancer progression, including proliferation, epithelial–mesenchymal transformation, senescence, multidrug resistance, and the maintenance of stem cell pluripotency^29^. To identify the mechanism by which LSD1 regulates stemness in LMP1-expressing BJAB cells, we performed QuantSeq 3′ mRNA-sequencing using lymphoma cells with or without LMP1 expression and/or LSD1 inhibition. From among the genes found to be decreased by LSD1 inhibition in LMP1-expressing cells, we selected stemness-related CHAC2^24^ for further study. CHAC2 was commonly reduced upon LSD1 inhibition in LMP1-expressing lymphoma cells, but not in control cells (Fig. 3A). This suggested that CHAC2 expression may be involved in the anticancer effects of TCP treatment or LSD1 inhibition. We then performed qPCR assays to verify the differential gene expression of CHAC2 and found that expression of LMP1 increased the mRNA expression of CHAC2, whereas knockdown of LSD1 decreased the mRNA expression of CHAC2 in LMP1-expressing BJAB cells and EBV-positive Raji cells (Fig. 3B). The mRNA expression level of CHAC2 was also dose-dependently decreased by TCP in both cell lines (Fig. 3C). This suggested that LMP1-induced CHAC2 expression is regulated by LSD1. When investigating the roles of CHAC2 in our setting, we found that CHAC2 knockdown negatively affected the stemness-associated properties of LMP1-expressing cells, as shown by colony formation, spheroid formation (Fig. 4A, Supple Fig. 2B), and ALDEFLUOR assays (Fig. 4B). Conversely, the colony forming ability of LMP1-expressing cells was upregulated by CHAC2 overexpression (Fig. 4C). We further found that CHAC2 overexpression induced that of SOX2, whereas CHAC2 knockdown inhibited SOX2 expression in LMP1-expressing cells (Fig. 4D). Moreover, in EBV-positive Raji cells, we observed significant decreases in SOX2 and NANOG under CHAC2 inhibition. Collectively, our data indicate that CHAC2 is required for stemness in LMP1-expressing cells, and that targeting CHAC2 and LSD1 can be effective approaches for suppressing the stemness of EBV-positive B cell lymphoma cells.

## Discussion

EBV-positive diffuse large B-cell lymphoma is a rare and aggressive B-cell lymphoma subtype in which most of the cancer cells are affected by EBV^2^. Recent studies involving mutation analysis, signal pathway analysis, and cancer stem cell-based experiments have explored the molecular pathogenesis of EBV positive DLBCLs with the goal of informing novel therapeutic strategies^3,4,30,31^. Here, we used an epigenetic modifier screen to identify target genes and functions that undergo epigenetic regulation in EBV positive DLBCLs. More specifically, we searched for drugs that could inhibit the cell growth of doxorubicin-treated LMP1-expressing B cell lymphoma cells. Based on the obtained results, we selected the LSD1 inhibitor, TCP, for further study. We verified the combined effect in LMP1-expressing B cell lymphoma cells and found that combined treatment with doxorubicin and TCP enhanced the level of apoptosis in LMP1-expressing cells beyond that seen for either mono-treatment. Other recent studies have observed increased apoptosis when screening drug pairs for induction of synergistic effects by an epigenetic modifier^27,32–36^. LSD1 has been found in diverse cancers and shows close relationships with many cellular processes, including cell proliferation, cell differentiation, stem cell biology, and malignant transformation^37^. Many studies have sought to elucidate mechanisms and inhibitory targets for LSD1, and examine how it induces stemness^5,6,10,13,15,16,18,20,29,33,38–41^. LSD1 has been shown to act as a co-repressor or co-activator, depending on the target: previous papers, its interactions with CoREST and NuRD lead to demethylation of monomethyl and dimethyl histone H3 lysine 4 (H3K4me1 and H3K4me2) and eventual repression of transcription, whereas its interactions with nuclear androgen receptor (AR) and estrogen receptor (ER) lead to demethylation of monomethyl and dimethyl histone H3 lysine 9 (H3K9me1 and H3K9me2) and eventual activation of transcription^39^. Here, LMP1 expression was found to decrease both H3K4me2 and H3K9me2, which suggests that LSD1 activity promotes demethylation in our setting. This also suggests that both transcription activator and repressor functions occur through the demethylation of H3K4me2 and H3K9me2. A previous study found that KDM6B (lysine demethylase 6B) was increased by LMP1, and functions to regulate neural stem cell differentiation by demethylating H3K27me3 as its substrate^42^. However, in the present study, H3K27me2 was shown to be increased by LMP1; this is presumed to reflect an increase in the activity of the histone methylase responsible for specifically methylating the H3K27me region. Here, we report that LMP1 expression induces the activity of LSD1 without altering its expression. Previous papers showed that LMP1 expression activates NF-κB signaling^43^ and NF-κB (p65) phosphorylates LSD1^44^, prompting us to speculate that the activity increase of LSD1 reflects its NF-κB-mediated phosphorylation by LMP1. The present results further suggest that the LMP1-mediated enhancement of LSD1 activity increases stemness, whereas the inhibition of LSD1 activity increases apoptosis in response to doxorubicin. In an effort to identify apoptosis-related genes induced to enable this effect, we performed a cDNA microarray analysis. Our results revealed that MMP9 and ROR2 were suppressed by LMP1 expression in DLBCLs, but were significantly increased by combined treatment of these cells with TCP plus doxorubicin. This suggests that the combined treatment upregulates MMP9 and ROR2 to induce apoptosis. Conversely, siRNA-mediated knockdown of MMP9 and ROR2 increased the stemness of LMP1-expressing cells. These results are consistent with previous reports that MMP9 regulates human cardiac stem cell death by upregulating apoptosis and ROR2 induces cell apoptosis via activating IRE1α/JNK/CHOP pathway in high-grade serous ovarian carcinoma^45,46^. To further understand the synergistic effect of TCP in this combined treatment, we explored the downstream targets of LSD1. We found that CHAC2 was downregulated by both TCP treatment and LSD1 knockdown. We also found that the stemness of LMP1-expressing cells is associated with the expression of CHAC2, as shown by the increase in SOX2. These results are consistent with previous reports that CHAC2 is critical for the self-renewal and maintenance of human embryonic stem cells^24^. In sum, we herein report for the first time that LMP1 upregulates LSD1 activity and stemness through CHAC2. Further studies including in vivo experiments on LSD1 and/or CHAC2 are needed to support and expand upon our present results. Based on our findings, we suggest that LSD1 and CHAC2 could be effective therapeutic targets for EBV-positive B cell lymphoma.

## Materials and methods

### Cell culture and treatment

The BJAB cell line was kindly provided by Dr. H. Y. Yoo (Sungkyunkwan University, Seoul, Korea). The Raji cell line was kindly provided by Dr. D. H. Nam (Samsung Medical Center, Seoul, Korea). The Riva cell line was purchased from Leibniz-Institut DSMZ-Deutsche Sammlung von Mikroorganismen und Zilkulturen GmbH (Braunschweig, Germany). BJAB, Riva, and Raji cells were cultured in RPMI-1640 medium supplemented with 10% heat-inactivated FBS, penicillin, and streptomycin (Gibco-BRL, Grand Island, NY, USA). The cells were incubated in a humidified 5% CO_2_ atmosphere. All cell lines were tested for mycoplasma and characterized by STR profiling as indicated in the DSMZ online. To establish LMP1-expressing stable cell lines, BJAB or Riva cells were transfected with EGFP-N1 control vector or EGFP-N1 + LMP1 vector via electroporation (Lonza, Cologne, Germany) and selected for 1 month with 1 mg/ml G418 (Sigma-Aldrich, Poole, UK). Small interfering RNAs (siRNAs) were purchased from Bioneer (Daejeon, South Korea). Cells were transiently transfected using Lipofectamine RNAiMAX (Invitrogen, Carlsbad, CA, USA). The CHAC2 expression and pCMV6-entry control vector were purchased from Origene (Rockville, MD, USA) and transfected into cells by electroporation. Doxorubicin and TCP were purchased from Selleckchem (Houston, TX, USA).

### Assessment of cell viability

The effects of the studied drugs and drug combinations on cell viability were monitored using Cell Counting Kit-8 reagent (CCK-8; Dojindo Laboratories, Kumamoto, Japan). Briefly, cells were incubated for 48 h at 37 °C in triplicate in a 96-well plate (final volume, 0.1 ml) in the presence or absence of the indicated drugs, and then 10 μl of CCK-8 reagent was added to each well. After a 2 h incubation at 37 °C, optical density (OD) at 450 nm was measured using a 96-well multiscanner autoreader. Cell viability was expressed as a percentage (OD of the experimental sample/OD of control).

### Soft agar colony formation and spheroid formation assays

Soft agar colony-formation assays were performed by first seeding cells in six-well plates (1 × 10^4^ cells/well) in a top layer of 0.4% agar-RPMI-FBS that was layered over a bottom layer of 0.8% agar-RPMI-FBS. Cultures were maintained at 37 °C. On day 14, cells were fixed with pure ethanol containing 0.05% crystal violet. Colonies containing at least 50 cells were counted and imaged with a Gel Doc XR+ system (Bio-Rad, Hercules, CA, USA). Spheroid formation assays were performed using a Cultrex UltiMatrix Reduced Growth Factor Basement Membrane (R&D Systems, Minneapolis, MN, USA). Cells were seeded in six-well plates (1 × 10^4^ cells/well) and grown for 10 days, and spheroids were observed through a microscope.

### RNA extraction and quantitative reverse transcription (RT)-PCR

Total RNA was prepared using a Qiagen RNA extraction kit (Qiagen, Valencia, CA, USA), according to the manufacturer’s instructions. For reverse transcription, 1 μg RNA was treated with RNase-free DNase and reverse transcribed to cDNA using an Omniscript RT kit (Qiagen). Real-time PCR was performed using primers specific for ROR2, MMP9, CHAC2, SOX2, and NANOG. Glyceraldehyde 3-phosphate dehydrogenase (GAPDH) was amplified as the control. The sequences of the primers were as follows: ROR2 forward, 5′-GTACGCATGGAACTGTGTGACG-3′ and reverse, 5′-AAAGGCAAGCGATGACCAGTGG-3′; MMP9 forward, 5′-GCCACTACTGTGCCTTTGAGTC-3′ and reverse, 5′-CCCTCAGAGAATCGCCAGTACT-3′; GAPDH forward, 5′-TGC ACC ACC AAC TGC TTA GC-3′ and reverse, 5′-GGC ATG GAC TGT GGT CAT GAG-3′. CHAC2 Hs00378072_m1, SOX2 Hs04234836_s1, NANOG Hs02387400_g1 and GAPDH Hs02786624_g1. These primers were purchased from Thermo Scientific (Waltham, MA, USA). Total RNA levels were used as an internal control to normalize the mRNA level, and fold-changes were calculated using the ∆∆Ct method.

### Western blot analysis

The utilized primary antibodies were specific for dimethyl histone H3 (Lys 4), dimethyl histone H3 (Lys 9), dimethyl histone H3 (Lys 27), histone H3, LSD1, MCL-1 (all from Cell Signaling, Beverly, MA, USA), LMP1 (Abcam, Cambridge, MA, USA), and GAPDH (Santa Cruz Biotechnology, Santa Cruz, CA, USA. The secondary antibody was HRP-conjugated goat anti-rabbit IgG (Santa Cruz Biotechnology). β-Actin (mouse monoclonal antibody, A5441; Sigma, St. Louis, MO, USA) was detected as a loading control. All primary antibodies were diluted 1:1000 and the secondary antibody was diluted 1:3000.

### Epigenetic modifier screening and cell viability assay

The epigenetic modifier library used in this study was kindly provided by the Korea Chemical Bank (http://www.chembank.org) of the Korea Research Institute of Chemical Technology. BJAB_vector, BJAB_LMP1, Riva_vector, and Riva_LMP1 cells were seeded in 96-well plates at 10,000 cells per well and incubated with doxorubicin (BJAB, 30 nM; Riva, 100 nM) with or without an epigenetic modifier (0.3 μM) at 37 °C in a humidified 5% CO_2_ incubator for 2 days. Cell viability was analyzed by CCK-8 assay as described above. Viable cells were also determined using a trypan blue exclusion assay. Briefly, cells were suspended in 0.4% trypan blue (1:1), loaded to a hemocytometer, and counted. The calculated percentage of unstained cells was taken as the percentage of viable cells.

### QuantSeq 3′ mRNA-sequencing

To examine genes whose expression was altered under LSD1 inhibition, cells were prepared, an oligo-dT primer containing an Illumina-compatible sequence at its 5′ end was hybridized to the RNA, and reverse transcription was performed. The RNA template was degraded, second-strand synthesis was initiated with a random primer containing an Illumina-compatible linker sequence at its 5′ end, and the obtained double-stranded library was purified with magnetic beads. The library was amplified to add the complete adapter sequences required for cluster generation. The finished library was purified and high-throughput single-end 75 sequencing was performed using a NextSeq 550 (Illumina, Inc., San Diego, CA, USA).

### Immunofluorescence staining

LMP1-expressing BJAB and Riva stable cells were washed with PBS, fixed in methanol for 10 min, incubated with blocking solution (3% BSA in PBS) for 1 h, and then incubated with anti-LMP1 (Abcam) and a fluorescence marker-conjugated secondary antibody (Molecular Probes, Eugene, OR, USA). Nuclei were stained with 4′, 6-diamidino-2-phenylindole (DAPI), and coverslips were mounted and analyzed using a confocal laser-scanning microscope (BX51; Olympus, Tokyo, Japan) and the appropriate software.

### Apoptosis assay

Apoptosis was detected using an Annexin-V-fluorescein isothiocyanate (FITC) Apoptosis Detection Kit (BD Biosciences, San Jose, CA, USA) and a BD FACSVerse flow cytometry system (BD Biosciences). Control and LMP1-expressing cells were exposed to doxorubicin, harvested, and processed according to the manufacturer’s instructions.

### ALDEFLUOR activity assay

The ALDEFLUOR assay was performed per the manufacturer’s instructions (StemCell Technologies, Vancouver, BC, Canada). Data acquisition was performed using a BD FACSVerse flow cytometry system (BD Biosciences).

### LSD1 activity assay

Nuclear extracts were prepared using NE-PER™ Nuclear and Cytoplasmic Extraction Reagents (Thermo Scientific, Waltham, MA, USA). LSD1 activity was detected using a KDM1/LSD1 Activity Quantification Assay Kit (Abcam) and a 96-well multiscanner autoreader.

### Statistical analysis

The results of this study are presented as the mean ± standard deviation; n = 3. P-values were determined by the Student’s *t*-test.

### Supplementary Information

Supplementary Figures.

## Data Availability

The datasets used and/or analyzed in the current study are available from the corresponding author upon reasonable request.
