# Supplementary material for: Lysine demethylase LSD1 is associated with stemness in EBV-positive B cell lymphoma
Source: Sci Rep. 2024 Mar 21;14:6764. doi: 10.1038/s41598-024-55113-6 (PMC10957933; doi:10.1038/s41598-024-55113-6)

Lysine demethylase LSD1 is associated with stemness in EBV-positive B cell lymphoma

Joo Hyun Kim[1], Chaehwa Park[2] and Won Seog Kim[1, 3,*]

[1]Department of Health Sciences and Technology, Samsung Advanced Institute for Health Sciences and Technology, Sungkyunkwan University, Seoul 06351, Korea

[2] Research Institute for Future Medicine, Samsung Medical Center, Sungkyunkwan University School of Medicine, Seoul 06351, Korea

[3]Division of Hematology and Oncology, Department of Medicine, Samsung Medical Center, Sungkyunkwan University School of Medicine, Seoul 06351, Korea

*Correspondence: Professor Won Seog Kim, Division of Hematology and Oncology, Department of Medicine, Samsung Medical Center, Sungkyunkwan University School of Medicine, 50 Irwon-dong, Seoul 06351, Korea; e-mail: wskimsmc@skku.edu (Tel: +82 2 3410 6548; fax: +82 2 3410 1757)

Supplementary Figure 1. Confirmation of stable LMP1-expressing BJAB and Riva cells. LMP1 (green) was detected by immunofluorescence staining. DAPI (blue) was used to stain cell nuclei. More than 80-90% of all cells expressed LMP1.

Supplementary Figure 2. Spheroid formation by knockdown of LSD1 or CHAC2 in LMP1-expressing BJAB cells. (A, B) Spheroid formation assays were performed using LMP1-expressing BJAB cells transfected with siCTL or siLSD1 (A) or siCHAC2 (B). After 10 days, spheroids were observed under a microscope. Scale bars : 100 μm. Bar graph presents spheroids/well compared to LMP1-expressing BJAB cells transfected with siCTL. The spheroid diameter was averaged by selecting 10 - 20 spheroids each cell lines. P-values were determined by the Student's *t*-test.

Supplementary Figure 3. Effect of Doxorubicin and TCP on LMP1-expressing BJAB cells. (A) Sensitivity of control or LMP1-expressing BJAB and Riva cells to TCP alone, doxorubicin alone, or TCP plus doxorubicin. Cell viability (left) and the CI (right) are shown for each cell line. Fa, fraction affected. (B) Representative scatter plots of PI (y-axis) vs annexin V (x-axis). (C) The protein levels of MCL-1 in cells treated as indicated were determined by Western blot analysis. Proteins that were transferred onto the membrane were sliced horizontally according to the molecular weights of the specific proteins of interest. In cases where there were closely spaced targeted proteins, the membrane was stripped, and a fresh round of probing was performed using primary antibodies from a different species source.

Supplementary Figure 4. Differential expression according to combined treatment of control or LMP1-expressing BJAB cells with TCP plus doxorubicin. (A) Cells were treated with TCP and doxorubicin for 72 h and cDNA microarray analysis was performed. Venn diagram shows the number of genes upregulated in both control and LMP1-expressing BJAB cells after combined treatment with TCP and doxorubicin. KEGG pathway analysis revealed that the combined treatment significantly increased genes involved in the release of cytochrome c from mitochondria. (B) The mRNA expression levels of MMP9 and ROR2 in BJAB cells versus untreated control cells were assessed by qRT-PCR and calculated using the △△Ct method. P-

values were determined by the Student's *t*-test. (C) Colony-formation assays were performed using LMP1-expressing BJAB cells transfected with the indicated siRNAs and treated with doxorubicin plus TCP. The data are presented as the mean ± standard deviation; n =3. Bar graph presents colony (%) compared to untreated cells. P-values were determined by the Student's *t*-test.

Supplementary Figure 1.

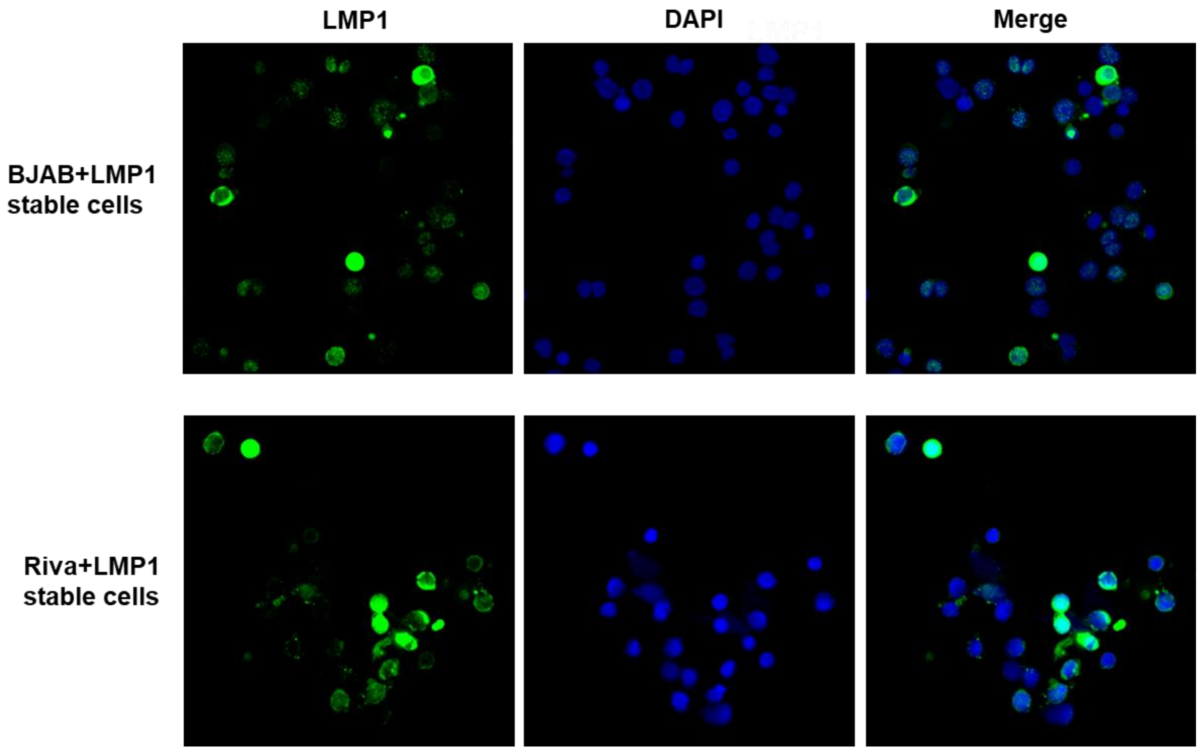

Supplementary Figure 2.

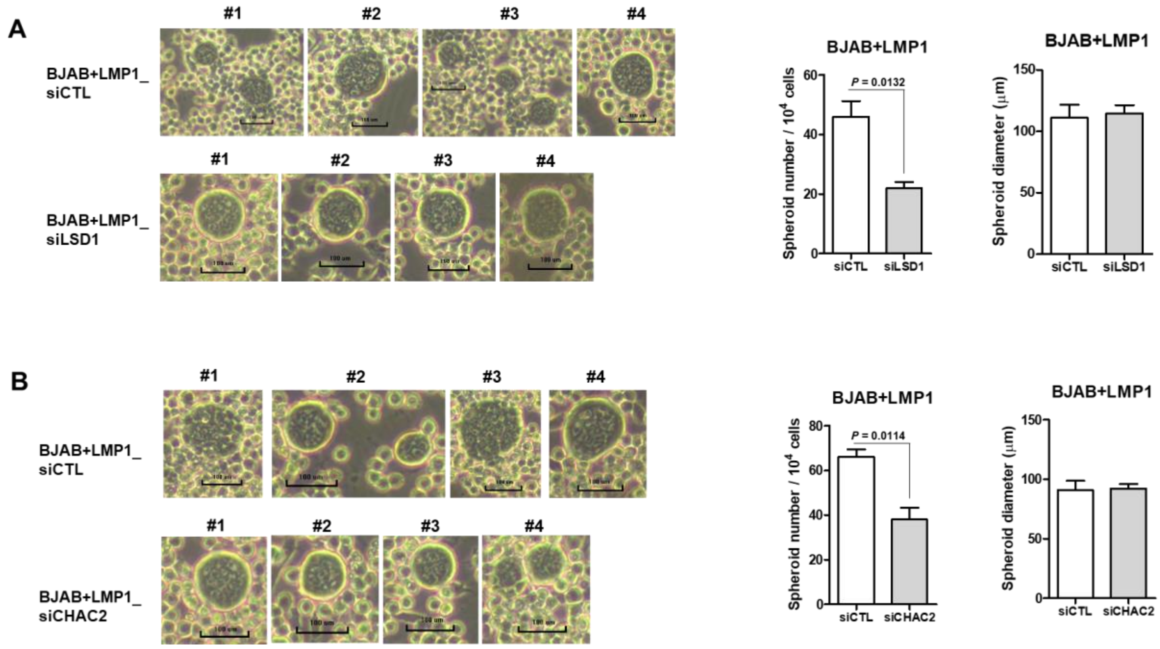

Supplementary Figure 3.

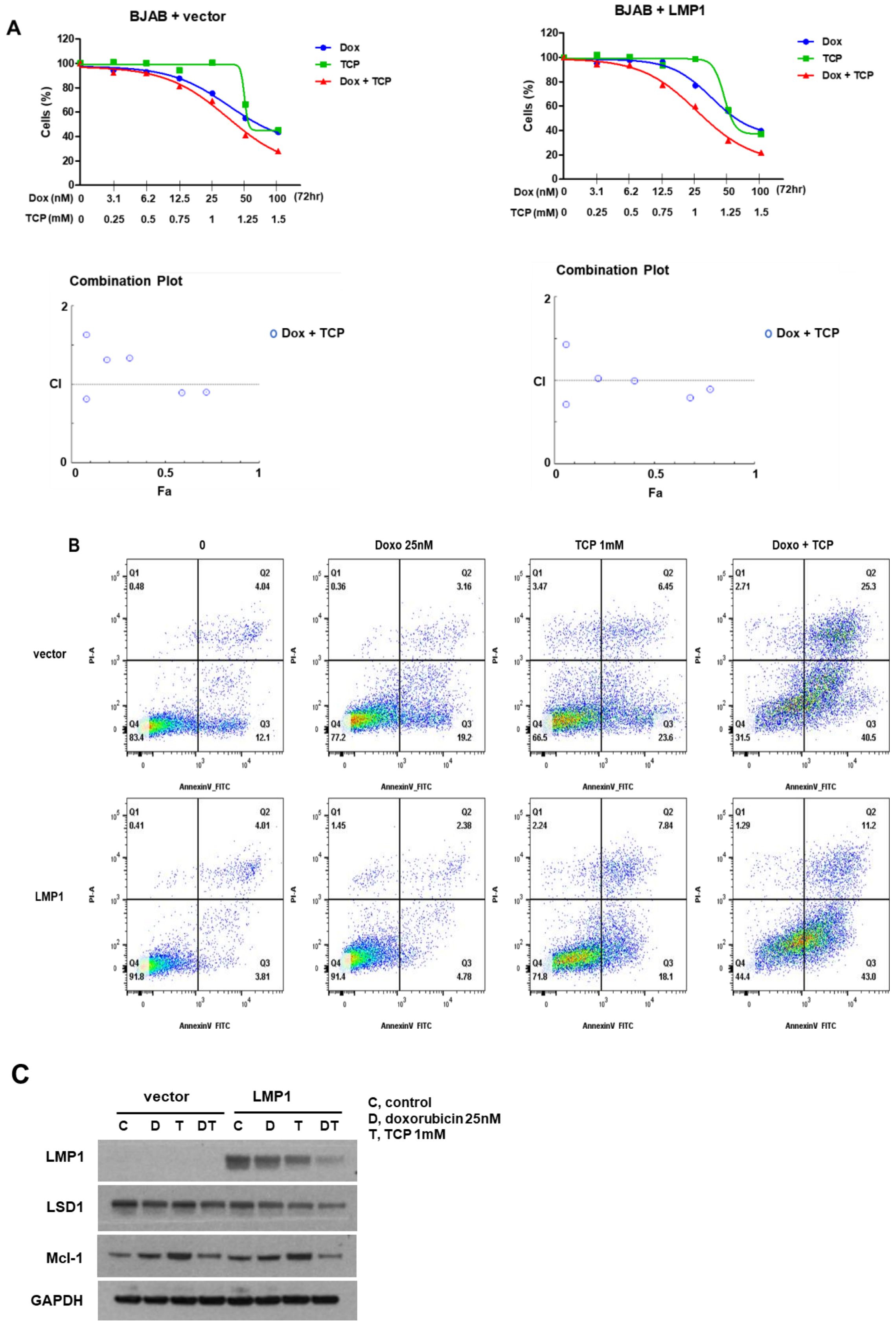

Supplementary Figure 4.

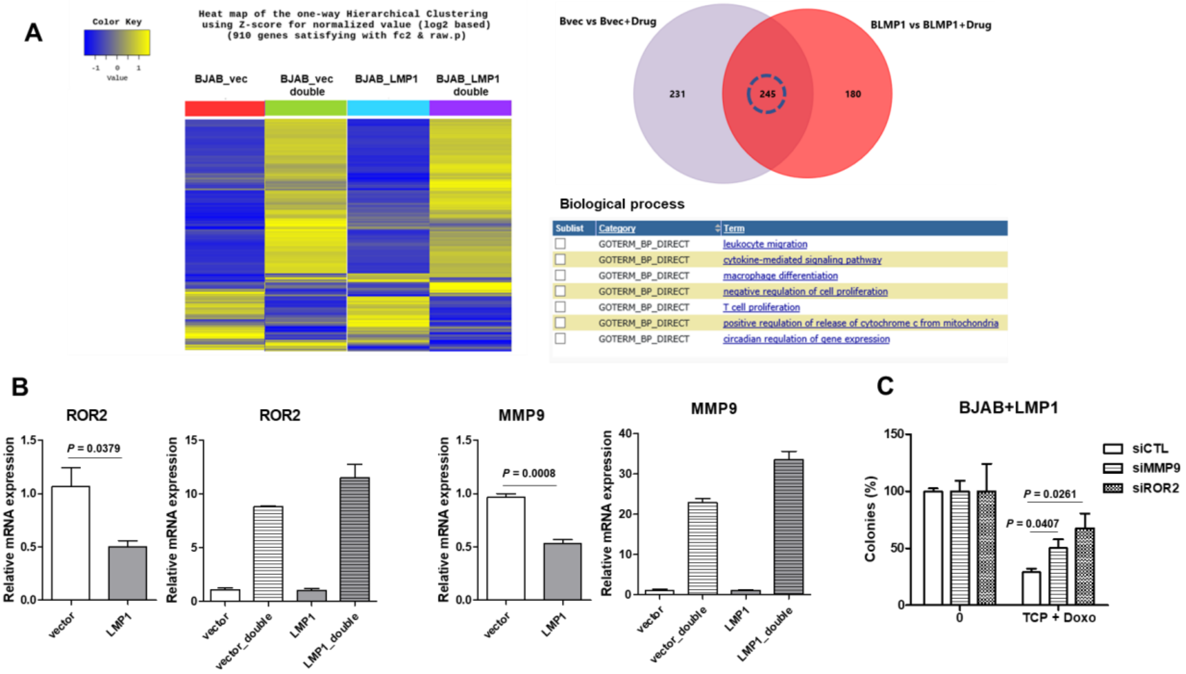

Original Western blot data

Figure 1C

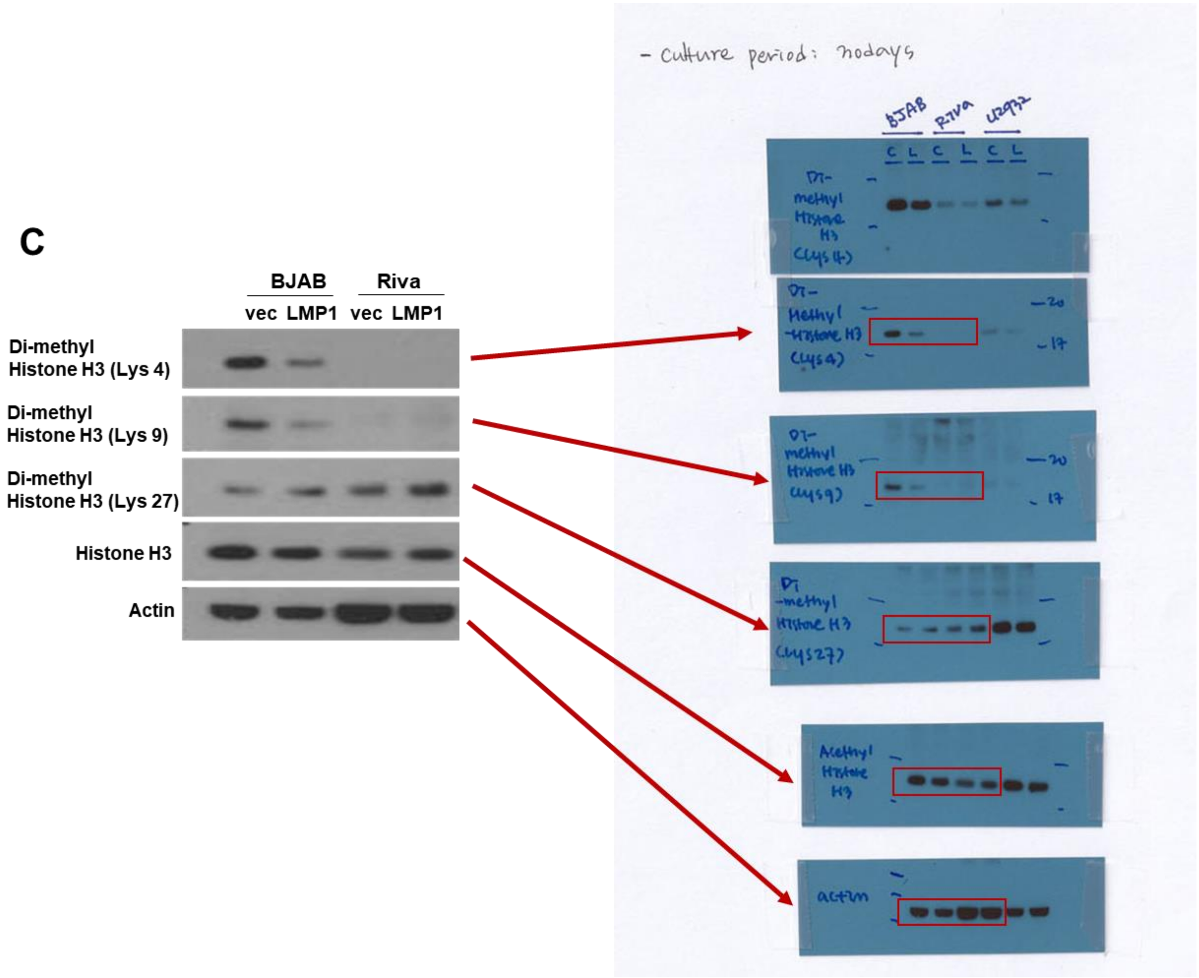

Figure 1D

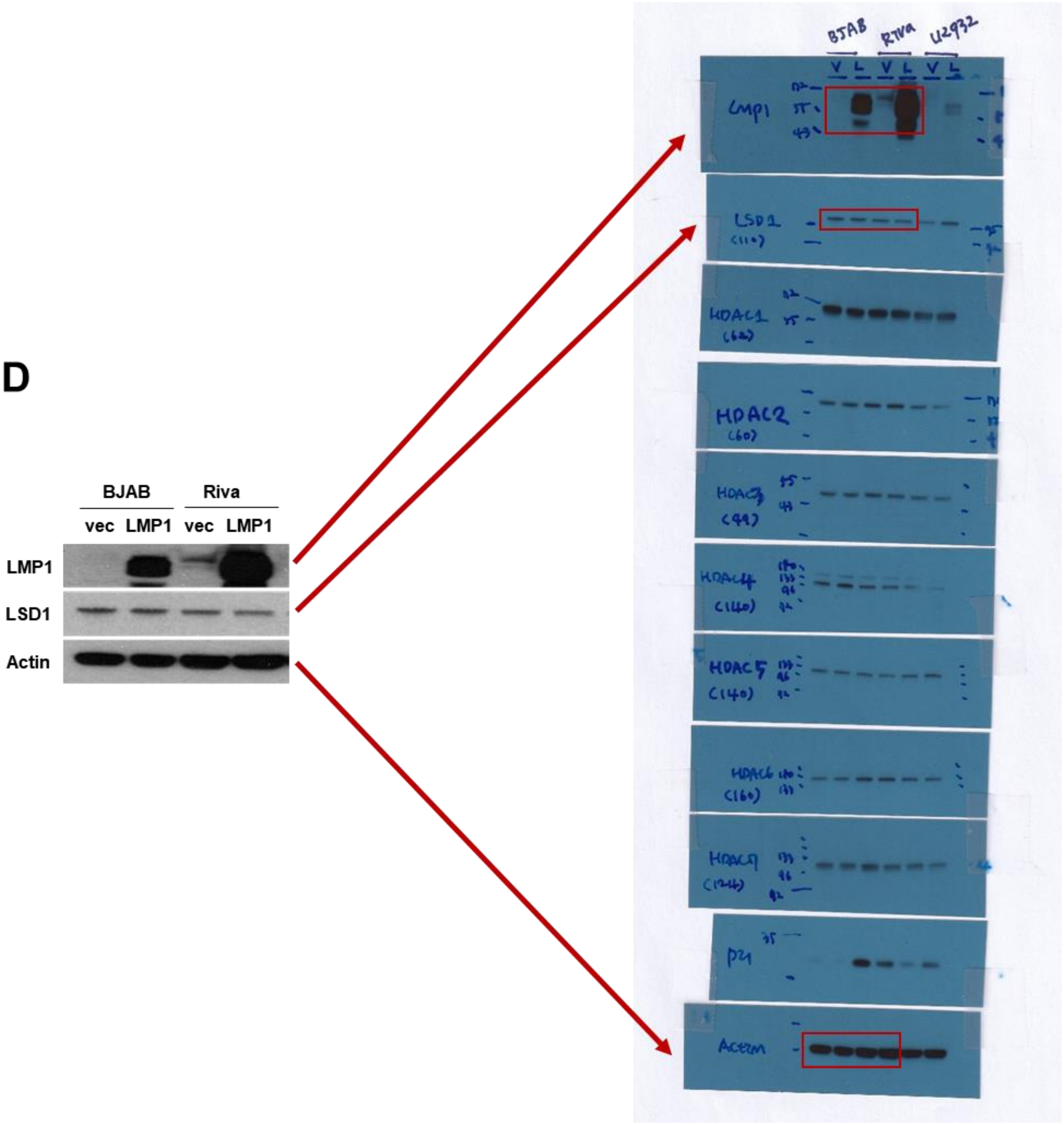

Supplementary Figure 3C

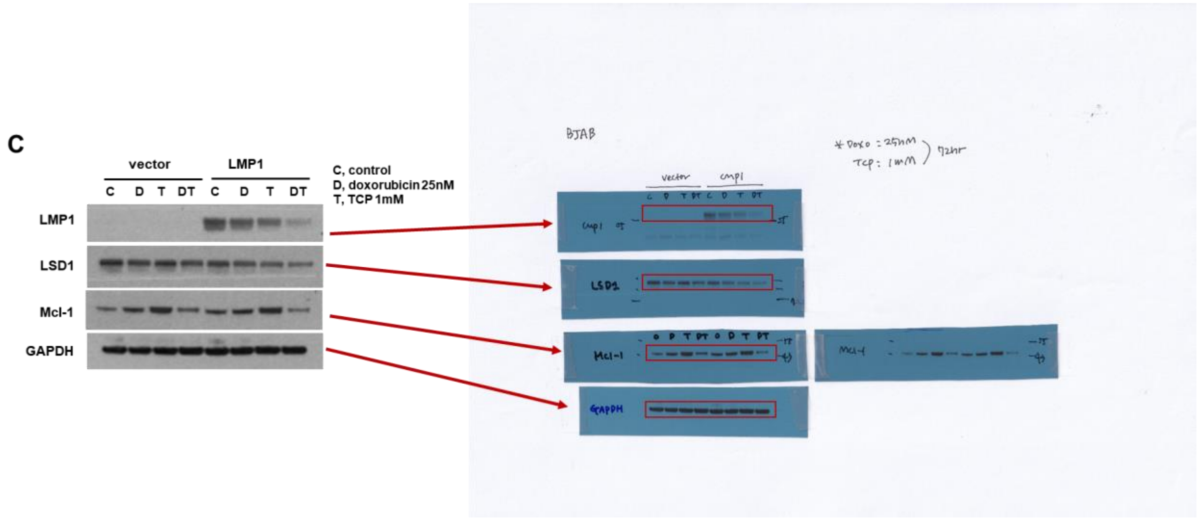

Supplement: Supplementary file 1 — Supplementary Figures. [file 41598_2024_55113_MOESM1_ESM.pdf]
